# Tiny noise, big mistakes: adversarial perturbations induce errors in brain–computer interface spellers

**DOI:** 10.1093/nsr/nwaa233

**Published:** 2020-09-10

**Authors:** Xiao Zhang, Dongrui Wu, Lieyun Ding, Hanbin Luo, Chin-Teng Lin, Tzyy-Ping Jung, Ricardo Chavarriaga

**Affiliations:** Ministry of Education Key Laboratory of Image Processing and Intelligent Control, School of Artificial Intelligence and Automation, Huazhong University of Science and Technology, Wuhan 430074, China; Ministry of Education Key Laboratory of Image Processing and Intelligent Control, School of Artificial Intelligence and Automation, Huazhong University of Science and Technology, Wuhan 430074, China; School of Civil Engineering and Mechanics, Huazhong University of Science and Technology, Wuhan 430074, China; School of Civil Engineering and Mechanics, Huazhong University of Science and Technology, Wuhan 430074, China; Centre of Artificial Intelligence, Faculty of Engineering and Information Technology, University of Technology Sydney, Sydney, NSW 2007, Australia; Swartz Center for Computational Neuroscience, Institute for Neural Computation, University of California San Diego, La Jolla, CA 92093, USA; Center for Advanced Neurological Engineering, Institute of Engineering in Medicine, University of California San Diego, La Jolla, CA 92093, USA; ZHAW DataLab, Zürich University of Applied Sciences, Winterthur 8401, Switzerland

**Keywords:** electroencephalogram, brain-computer interfaces, BCI spellers, adversarial examples

## Abstract

An electroencephalogram (EEG)-based brain–computer interface (BCI) speller allows a user to input text to a computer by thought. It is particularly useful to severely disabled individuals, e.g. amyotrophic lateral sclerosis patients, who have no other effective means of communication with another person or a computer. Most studies so far focused on making EEG-based BCI spellers faster and more reliable; however, few have considered their security. This study, for the first time, shows that P300 and steady-state visual evoked potential BCI spellers are very vulnerable, i.e. they can be severely attacked by adversarial perturbations, which are too tiny to be noticed when added to EEG signals, but can mislead the spellers to spell anything the attacker wants. The consequence could range from merely user frustration to severe misdiagnosis in clinical applications. We hope our research can attract more attention to the security of EEG-based BCI spellers, and more broadly, EEG-based BCIs, which has received little attention before.

## INTRODUCTION

A brain–computer interface (BCI), which has been extensively used in neuroscience, neural engineering and clinical rehabilitation, offers a communication pathway that allows people to interact with computers using brain signals directly [1–4]. There are many approaches to collecting signals from the brain. Electroencephalogram (EEG), usually measured from the scalp, may be the most popular one due to its simplicity and low cost [5].

**Figure 1.**
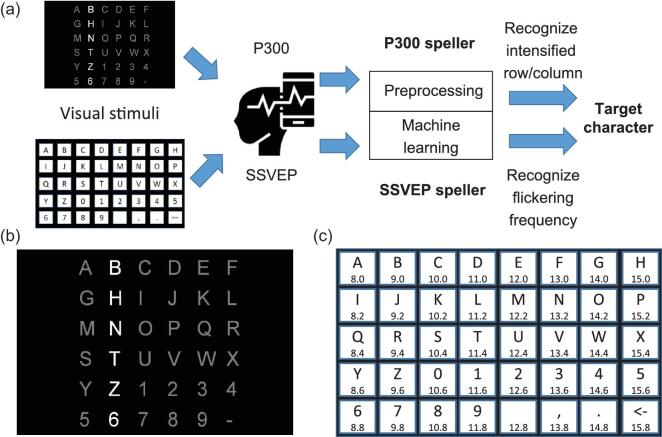
A P300 speller and an SSVEP speller. (a) Workflow of a P300 speller (top path) and an SSVEP speller (bottom path). For each speller, the user watches the stimulation interface, focusing on the character he/she wants to input, and EEG signals are recorded and analyzed by the speller. The P300 speller first identifies the row and the column that elicit the largest P300, and then outputs the character at their intersection. The SSVEP speller identifies the output character directly by matching the user’s EEG oscillation frequency with the flickering frequency of each candidate character. (b) Stimulation interface of a P300 speller, where the second column is intensified. (c) Stimulation interface of an SSVEP speller. The number below each character indicates its flickering frequency (Hz).

An EEG-based BCI speller allows a user to input text to a computer by thought [6,7]. It enables people with severe disabilities, e.g. amyotrophic lateral sclerosis (ALS) patients, to communicate with computers or other people. The two main types of EEG-based BCI spellers are P300 spellers [6] and steady-state visual evoked potential (SSVEP) spellers [7], which elicit different EEG patterns, as illustrated in Fig. 1(a).

A P300 speller, which uses P300 evoked potentials as its input signal [8], was first invented by Farwell and Donchin in 1988 [6] and further developed by many others [9–12]. P300 is a positive deflection in voltage, typically appearing around 250 to 500 ms after a rare target stimulus occurs [13]. It is an endogenous potential linked to people’s cognitive processes, such as information processing and decision making [14,15]. The standard oddball paradigm is usually used to elicit P300, in which rare target stimuli are mixed with high-probability nontarget ones. The P300 speller considered in this article uses a 6 × 6 character matrix, which consists of 26 letters and 10 other symbols, as shown in Fig. 1(b). The user stares at the character he/she wants to input, while a row or column is rapidly intensified sequentially. The corresponding EEG signals are recorded and classified as a target (containing P300) or nontarget (not containing P300) for each intensification. Then, the computer identifies the character at the intersection of the target row and the target column, which elicit the largest P300s, as the output. For reliable performance, each row and column may have to be intensified multiple times, which reduces the speed of the P300 speller.

Compared with the P300 speller, an SSVEP speller has the advantages of a high information transfer rate (ITR), little user training and some immunity to artifacts [16–18]. When the user stares at a visual target flickering at a specific frequency, usually between 3.5 and 75 Hz, electrical signals of the same frequency, as well as its corresponding harmonics, can be observed from the EEG signals [16]. In an SSVEP speller, the pictures of different characters are flickering at different frequencies, so that a classifier can directly identify the output character from a large number of candidates by matching their flickering frequencies with the user’s EEG oscillation frequency. Since all characters in an SSVEP speller are flickering simultaneously (in contrast to sequential intensification in a P300 speller), they can have much higher ITRs. The SSVEP speller considered in this study has 40 characters (Fig. 1(c)), whose stimulation frequencies are from 8 to 15.8 Hz with 0.2 Hz increment [19].

Machine learning is used in BCI spellers to construct the classifiers to detect the brain responses to stimuli (i.e. the P300 or SSVEP patterns). Most studies so far focused on making the BCI classifiers faster and more reliable; however, few have considered their security. It has been found in other application domains that adversarial examples [20], which are normal examples contaminated by deliberately designed tiny perturbations, can easily fool machine learning models. These perturbations are usually so small that they are indistinguishable to human eyes. Existing studies on adversarial examples focused largely on deep learning models for computer vision. For example, it was found that a picture of a panda, after adding a weak adversarial perturbation, can be misclassified as a gibbon by a deep learning classifier [21]. Kurakin *et al.* [22] found that printed photos of adversarial examples can degrade the performance of an ImageNet Inception classifier. Athalye *et al.* [23] three-dimensionally printed a turtle with an adversarial texture, which was classified as a riffle from almost every viewpoint. Recently, adversarial examples were also found in traditional machine-learning models [24] and in many other application domains, e.g. speech recognition [25], text classification [26], malware identification [27], etc. Because of the high risk of adversarial attacks, many defense mechanisms have been proposed, such as defensive distillation [28], adversarial training [21,29,30] and so on [31–33]. However, these approaches only improve empirical adversarial robustness, which is not certified and may be broken by a stronger attack approach [34,35]. Recently, researchers started to investigate provable guarantees of adversarial robustness, yet there is still a huge gap between certified robustness and empirical robustness [36–39].

This article aims to expose a critical security concern in EEG-based BCI spellers, and more broadly, EEG-based BCIs, which has received little attention before. It shows for the first time that one can generate tiny adversarial EEG perturbation templates for target attacks for both P300 and SSVEP spellers, i.e. mislead the classification to any character the attacker wants, regardless of what the user’s intended character is. The consequence could range from merely user frustration to severe misdiagnosis in clinical applications [40]. We believe a new and more detailed understanding of how adversarial EEG perturbations affect BCI classification can inform the design of BCIs to defend against such attacks.

There have been some studies on adversarial attacks of time-series signals [25,40–42]. They treated time-series signals just like images, and then applied essentially the same attack approaches in image classification to generate adversarial perturbations. As a result, they need to know the full time series before computing the adversarial perturbations, which means that these approaches are not causal and hence cannot be implemented in real-world applications. For example, to attack a voice command, previous approaches need to record the entire voice command first, and then design the perturbation. However, once the perturbation is obtained, the voice command has already been sent out (e.g. to a smartphone or Amazon Echo), so there is no chance to add the perturbation to the voice command to actually perform the attack.

What distinguishes the attack approaches in this article most from previous ones is that it explicitly considers the causality in designing the perturbations. The adversarial perturbation template is constructed directly from the training set and then fixed. So, there is no need to know the test EEG trial and compute the perturbation specifically for it. The perturbation can be directly added to a test EEG trial as soon as it starts, and hence satisfies causality and can be implemented in practice. Thus, it calls for an urgent need to be aware of such attacks and defend against them.

A closely related concept is universal adversarial perturbations [43], which can also be viewed as adversarial perturbation templates and have been used to attack deep learning models in image classification. This study focuses on the security of a traditional and most frequently used BCI pipeline, which consists of separate feature extraction and classification steps, whereas universal adversarial perturbations are usually designed for nontarget attacks of end-to-end deep learning models.

To summarize, our contributions are as follows.

- We show, for the first time, that tiny noise can significantly manipulate the outputs of P300 and SSVEP spellers, exposing a critical security concern in BCIs.
- Instead of deep learning models, we consider the classical BCI pipeline consisting of feature extraction and classification as our victim models, which dominate practical BCI spellers.
- Our generated adversarial perturbation templates satisfy the causality of time-series signals, which was rarely paid attention to before.

## RESULTS

### Performance evaluation

We used two measures to evaluate the performance of a BCI speller, the classification accuracy and the ITR [44], which measures the typing speed of the speller:

(1) \begin{eqnarray*} \mathrm{ITR}&=&\frac{1}{T}\Big[\log _2Q+R\log _2R\nonumber\\ &&\,\,\,\,\,\,+\,(1-R)\log _2\frac{1-R}{Q-1}\Big]. \end{eqnarray*}

Here *T* is the average time (minutes) spent to input a user character, *Q* is the number of different characters (which was 36 in our P300 speller and 40 in the SSVEP speller) and *R* is the classification accuracy. The unit of the ITR is bits/min. When the classification accuracy is lower than a random guess, i.e. *R* ≤ 1/*Q*, the ITR is directly set to 0.

To distinguish between the character the user wants to spell, and the character the attacker wants to mislead to, we denote the former ‘user character’ and the latter ‘attacker character’. Accordingly, ‘user score’ and ‘user ITR’ are used to describe the classification accuracy of user characters and the corresponding ITR, respectively. An ‘attacker score’ is defined as the ratio that the perturbation template leads the speller to output an attacker character, and the corresponding ‘attacker ITR’ is calculated by replacing *R* in equation (1) with the attacker score. A higher attacker score or attacker ITR represents a better target attack performance.

### Security of the P300 speller

#### Data information

We used a public P300 dataset (dataset II) introduced by Blankertz *et al.* [45]. It recorded 64-channel EEG signals from two subjects (A and B). The EEG data were sampled at 240 Hz, bandpass filtered to 0.1–40 Hz, then *z* normalized for each channel. There were 85 training character trials and 100 test character trials for each subject. For each trial, a set of 12 random intensifications (six rows and six columns) were repeated 15 times (i.e. each row was intensified 15 times, and each column was also intensified 15 times). Each intensification lasted for 100 ms, after which the character matrix was blanked for 75 ms. So, it took (100 + 75) × 12 × 15 = 31 500 ms, or 31.5 s, to input a character. The spelling speed can be improved by using fewer repeats, e.g. 10 or 5; however, the spelling accuracy generally decreases with a smaller number of repeats.

Note that all the following experiments were also successfully performed on a public ALS P300 dataset with eight ALS patients (see the online supplementary material for details).

#### The victim model

The victim model was a Riemannian geometry-based approach, which won the Kaggle BCI challenge (see https://www.kaggle.com/c/inria-bci-challenge) in 2015. First, 16 xDAWN spatial filters [46], eight for the target trials and another eight for the nontarget trials, were designed to filter all the trials. The template-signal covariance matrices of the EEG epochs were projected onto the tangent space of a Riemannian manifold [47–49], using an affine-invariant Riemannian metric as its distance metric. Finally, we classified the feature vectors with a logistic regression model in the tangent space. The details can be found in the online supplementary material. The model was trained with class-specific weights to accommodate class imbalance. All operations in these blocks are differentiable, so we reimplemented them using Tensorflow [50] to facilitate the gradient calculation.

To get the label (target or nontarget) of an intensification, an epoch between 0–600 ms from the beginning of the intensification was extracted and fed into the victim model to calculate the target probability. Because each row and column was intensified multiple times, voting was performed for each trial to get the target row and target column, and hence the target character.

#### Baseline performance

The first part of Table 1 shows the baseline performance of the clean EEG data (without adding any perturbations). As the number of intensification repeats increased, the user score increased, indicating that the classification accuracy of the user characters increased. Meanwhile, the user ITR decreased, because the time needed to input each character significantly increased.

**Table 1.** P300 speller attack results. Before attack: baselines on clean EEG data (without adding any perturbations) and Gaussian-noise-perturbed EEG data, and the corresponding SPRs (dB). After attack: average user/attacker scores/ITRs of the 36 attacker characters in target attacks and the corresponding period and trial SPRs (dB).

		Before attack					After attack					
		Clean		Gaussian noise			User		Attacker			
Subject	No. of repeats	Score	ITR	Score	ITR	SPR	Score	ITR	Score	ITR	Period SPR	Trial SPR
A	5	0.64	13.07	0.65	13.40	20.8	0.072	0.248	0.825	19.8	20.8	25.8
	10	0.85	10.62	0.84	10.40	21.0	0.049	0.052	0.900	11.7	21.0	25.9
	15	0.91	8.03	0.92	8.19	21.0	0.040	0.021	0.950	8.7	21.0	25.9
B	5	0.79	18.41	0.79	18.41	25.2	0.107	0.578	0.713	15.6	25.2	30.2
	10	0.91	11.96	0.89	11.50	25.5	0.061	0.093	0.860	10.9	25.5	30.4
	15	0.93	8.35	0.91	8.03	25.6	0.049	0.034	0.907	8.0	25.6	30.5

The second part of Table 1 shows the baseline performance when we added Gaussian noise to the raw EEG data, averaged over 10 runs. The Gaussian noise perturbations were preprocessed in the same way as the adversarial perturbations, by replacing the perturbation ($\widetilde{P}$ in equation (6)) with standard Gaussian noise, so that they had the same energy. We use a signal-to-perturbation ratio (SPR) to quantify the magnitude of the perturbation, which is also presented in the second part of Table 1. Gaussian noise perturbations had almost no impact on the user score and the user ITR at all, not to mention forcing the P300 speller to output a specific attacker character. These results suggest that more sophisticated adversarial perturbations are needed to attack the P300 speller.

#### Performance under adversarial attacks

**Figure 2.**
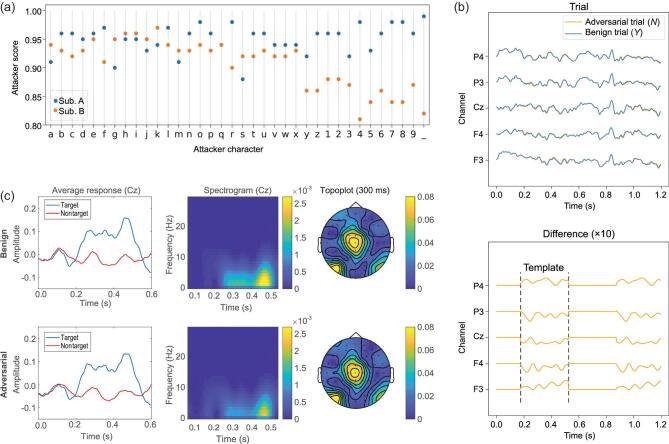
P300 speller attack results. (a) Attacker scores of manipulating the P300 speller to misclassify the 100 test character trials into a specific attacker character. The P300 speller used 15 intensification repeats for each character. (b) EEG trials before and after adversarial perturbations, which are almost completely overlapping (the SPRs are shown in Table 1), and the difference (magnified ten times) between the adversarial trial and the benign trial. The nonzero part of the difference is the adversarial perturbation template, which is added to a benign EEG trial according to the attacker character. The adversarial perturbation led the P300 speller to misclassify letter *Y* into *N*. (c) The left column shows the average of 100 × 15 × 2 = 3000 target trials (containing P300) and an average of 100 × 15 × 10 = 15 000 nontarget trials (not containing P300) in channel Cz for benign and adversarial trials; the middle column shows the spectrogram of the difference between the average target trial and the average nontarget trial in channel Cz for benign and adversarial trials; the right column shows the topoplot of the difference between the average target trial and the average nontarget trial for benign and adversarial trials. Panels (b) and (c) present the visualization of the adversarial perturbations for subject A.

We added the adversarial perturbation template to the test EEG trials to validate whether it was effective in misleading the P300 speller. In Fig. 2(a) we show the attacker scores of the 36 characters. The attacker can manipulate the P300 speller to spell whatever character he/she wants, regardless of what the user’s intended character is, with a higher than 90% average success rate.

The third part of Table 1 shows the average user scores and ITRs with different numbers of intensification repeats. The user scores and ITRs were close to zero, suggesting that the user almost cannot correctly input the character he/she wanted.

The fourth part of Table 1 shows the average attacker scores and ITRs with different numbers of intensification repeats. The attacker score increased with the number of intensification repeats, because more repeats increased the number of times that the attacker can inject the perturbation into the benign EEG trial.

To better quantify the magnitude of the perturbations, we also calculated two SPRs. The adversarial perturbation template was only added at some specific periods of the EEG trial, as shown in Fig. 2(b); therefore, we defined a ‘period SPR’ to measure the SPR of the perturbed period, and also a ‘trial SPR’ to measure the SPR of the entire trial. The last part of Table 1 shows these SPRs. They were higher than 20 dB, suggesting that the adversarial perturbation template may be undetectable when added to benign EEG trials.

#### Visualization of the adversarial perturbations

In addition to high attack performance, another requirement in adversarial attacks is that the perturbations should not be detected easily. In Fig. 2(b) we show a typical EEG trial before and after the adversarial perturbation on subject A. For clarity, we only show channels F3, F4, Cz, P3 and P4, which evenly distribute on the scalp. One can barely distinguish the adversarial EEG trial from the original EEG trial.

A traditional way to visualize the P300 signal is to take the average of multiple P300 trials. We also took this approach to check if there was a noticeable difference between the average target (or nontarget) trials, before and after perturbation. In Fig. 2(c) we show the results from the Cz channel. One can hardly observe any differences. In Fig. 2(c) we also show the spectrograms and topoplots of the difference between the average target EEG trial and the average nontarget EEG trial. The original and adversarial spectrograms (or topoplots) show very similar energy distributions and are hardly distinguishable by human eyes.

### Security of the SSVEP speller

#### Data information

The dataset was first introduced by Wang *et al.* [19] as a benchmark dataset for SSVEP-based BCIs. The 64-channel signals were recorded from 35 subjects using an extended 10–20 system. During the experiments, the subjects were facing a monitor, in which a 5 × 8 character matrix was flickering. Different flickering frequencies were assigned to the 40 characters, respectively, ranging from 8 to 15.8 Hz with 0.2 Hz increment, as shown in Fig. 1(c). Six blocks of EEG signals were recorded from each subject, each with 40 trials, corresponding to the 40 target characters. Each trial was downsampled to 250 Hz and lasted 6 s, including 0.5 s before stimulus onset, 5 s for stimulation and 0.5 s after stimulus offset.

Chen *et al.* [51] showed that an SSVEP at the stimulation frequency and its harmonics usually starts to be evoked with a delay around 130–140 ms; hence, we extracted EEG signals between [0.13, 1.38] s after the stimulus onset as the input to the victim model. Nine channels over the occipital and parietal areas (Pz, POz, PO3, PO4, PO5, PO6, Oz, O1 and O2) were chosen. The signals were bandpass filtered to 7–90 Hz with a fourth-order Butterworth filter.

#### The victim model

Extracting the frequency information of SSVEPs is an essential step in recognizing the stimulation frequency, and hence the user character. A natural solution is to utilize a fast Fourier transform to estimate the spectrum, so that the energy peaks can be matched to the stimulation frequency; however, canonical correlation analysis (CCA) was recently shown to be more promising in identifying the stimulation frequency [51,52]. Thus, CCA-based frequency recognition was used in the victim model.

CCA is a statistical approach that can be used to extract the underlying correlation between two multichannel time series [53]. Its main idea is to find a linear combination of channels for each time series, so that their correlation is maximized. When applied to SSVEP spellers, CCA is utilized to calculate the maximum correlation between the input EEG signals and a standard reference signal, which consists of the sinusoidal signal of a stimulation frequency and its (*N_q_* − 1) harmonics (*N_q_* = 5 in our case).

Mathematically, let $X\in \mathbb {R}^{N_e \times N_s}$ denote an EEG trial with *N_e_* channels and *N_s_* samples, and let *Y_f_* be a standard reference signal of stimulation frequency *f*. The (*c, n*)th entry of *Y_f_* is

(2) \begin{eqnarray*} Y_f(c,n) = \left\lbrace \begin{array}{@{}l@{\quad }l@{}} \sin \left((c+1) \pi \frac{f}{f_s} n\right), & c \text{ is odd,}\\ \cos \left(c \pi \frac{f}{f_s} n\right), & c \text{ is even,} \end{array}\right.\nonumber\\ \end{eqnarray*}

where *f_s_* is the sampling rate, 1 ≤ *c* ≤ 2*N_q_* and 1 ≤ *n* ≤ *N_s_*. To calculate the maximum correlation coefficient ρ(*X, Y_f_*), *X* and *Y_f_* are first *z* normalized, and then ρ(*X, Y_f_*) is computed as the square root of the largest eigenvalue of matrix

(3) \begin{eqnarray*} S(X,Y_f)=\left(XX^{T} \right)^{-1}XY_f^{T} \left(Y_fY_f^{T} \right)^{-1}Y_fX^{T},\nonumber\\ \end{eqnarray*}

i.e.

(4) \begin{eqnarray*} \rho (X, Y_f) = \sqrt{\lambda _{\max }\!\left(S(X,Y_f)\right)}. \end{eqnarray*}

More detailed derivations can be found in the online supplementary material.

Let $F=\lbrace f_i\rbrace _{i=1}^K$ be the set of *K* candidate stimulation frequencies (*K* = 40 in our case). Then, the SSVEP speller outputs the character corresponding to the following stimulation frequency:

(5) \begin{eqnarray*} f^* = \arg \max _{f\in F}\rho (X, Y_f). \end{eqnarray*}

#### Baseline performance

Among the 35 subjects, eight with the best baseline performances (shown in the first part of Table 2) were used in our experiments (the baseline performances of all 35 subjects can be found in Fig. 2 of the online supplementary material).

**Table 2.** SSVEP speller attack results. Before attack: baselines on clean data (without adding any perturbations), Gaussian-noise-perturbed EEG data and periodic-noise-perturbed EEG data. After attack: average user/attacker scores/ITRs of 40 attacker characters in target attacks and the corresponding SPRs (dB).

	Before attack							After attack				
	Clean		Gaussian noise		S/C periodic noise			User		Attacker		
Subject	Score	ITR	Score	ITR	Score	ITR	SPR	Score	ITR	Score	ITR	SPR
3	0.88	182.5	0.88	181.6	0.71/0.87	129.0/178.6	25.0	0.44	61.1	0.58	93.3	25.3
4	0.90	186.9	0.90	187.1	0.68/0.87	121.0/177.5	25.0	0.07	2.3	0.95	210.1	25.7
12	0.90	188.8	0.90	188.0	0.78/0.86	150.0/174.3	25.0	0.26	26.6	0.75	139.5	25.5
22	0.82	160.0	0.79	150.2	0.74/0.75	137.5/140.7	25.0	0.11	6.1	0.91	191.0	25.1
25	0.90	189.1	0.89	184.1	0.84/0.87	168.3/177.2	25.0	0.78	148.2	0.17	13.8	26.7
26	0.90	187.8	0.88	180.3	0.58/0.84	94.4/168.1	25.0	0.03	0.1	1.00	229.9	24.8
32	0.87	176.9	0.87	179.6	0.59/0.82	97.2/163.6	25.0	0.03	0.0	1.00	231.4	24.9
34	0.80	154.7	0.79	151.8	0.48/0.72	66.8/130.4	25.0	0.03	0.0	1.00	231.2	25.9

Because SSVEPs are highly susceptible to periodic noise, we evaluated the robustness of the victim model to Gaussian noise and sinusoidal noise of a random single frequency chosen from 40 stimulation frequencies, and a random phase chosen from −π/2 to π/2. We also considered compound sinusoidal noise that can be regarded as the summation of single sinusoidal noise of different frequencies, random amplitudes and random phases. The SPRs were all set to 25 dB, so that the energy of the Gaussian noise and single/compound (S/C) periodic noise was comparable to that of the adversarial perturbation templates. The ‘Gaussian noise’ and ‘S/C periodic noise’ panels of Table 2 show the results on these noisy data, averaged over 10 runs, respectively. The victim model was almost completely immune to the Gaussian noise. The single periodic noise degraded the model performance more than the Gaussian noise or compound periodic noise.

#### Performance under adversarial attacks

**Figure 3.**
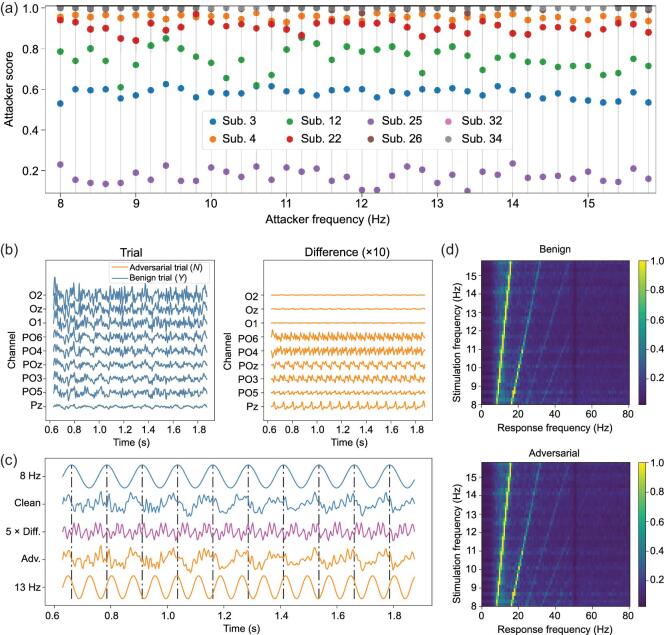
SSVEP speller attack results. (a) Attacker scores of manipulating the SSVEP speller to misclassify the 5 × 40 = 200 test character trials into a specific attacker frequency (character). (b) The left column shows the EEG trials before and after adversarial perturbations, for subject 26; the right column shows the difference (adversarial perturbation) between the adversarial EEG trial and the benign EEG trial for subject 26, magnified by ten times to make them visible. The adversarial perturbation led the SSVEP speller to misclassify the letter *Y* into *N*. (c) Detailed signal analysis for channel POz of subject 26. The clean signal was the average of all six trials of 8 Hz stimulation frequency, and the adversarial trial was the average of the same trials with δ_13Hz_ added. Standard 8 and 13 Hz sinusoidal signals are shown as references. The green dash–dot lines mark the 8 Hz periodicity. (d) Normalized spectra of SSVEPs for 40 stimulation frequencies, averaged over all the chosen channels and all 40 subjects.

We generated 40 adversarial perturbation templates, each forcing the SSVEP speller to output a specific character. In Fig. 3(a) we show their attacker scores. For six of the eight subjects, their output character can be manipulated to any character the attacker wanted, at 70%–100% success rate. Interestingly, due to individual differences, subjects 3 and 25 showed some resistance to adversarial perturbation templates.

The fifth and sixth parts of Table 2 show the averaged user and attacker performances, respectively. The adversarial perturbation templates were very effective on most subjects (except subjects 3 and 25), reducing both the user scores and the user ITRs to almost zero, i.e. the user almost cannot correctly input any character he/she wanted. The attacker scores for five subjects were close to one, i.e. the attacker was able to force the SSVEP speller to output any character he/she wanted. The SPRs were all around 25 dB, comparable to the SPRs for random noise.

#### Visualization of the adversarial perturbations

In this subsection we show the characteristics of the adversarial perturbation templates, and verify their imperceptibility to some widely used approaches for evaluating the quality of SSVEPs.

In Fig. 3(b) we show the EEG signals before and after adversarial perturbations, along with the magnified difference. The SSVEP speller misclassified the user character, which was supposed to be *Y* (8.6 Hz), into *N* (13.2 Hz). Human eyes can barely recognize the difference between the benign and the adversarial EEG trials. After being magnified by 10 times, the perturbation looks periodical, which can modify the user frequency to the attacker frequency.

We compared the clean and adversarial EEG signals with standard sinusoidal signals in Fig. 3(c), using subject 26 as an example. We took the average of the clean temporal waveforms of 8 Hz SSVEPs from channel POz, and did the same for their adversarial signals with δ_13Hz_ added (which forced the SSVEP speller to output the character of 13 Hz stimulation frequency). We chose channel POz because the adversarial perturbation on this channel had one of the largest amplitudes, as shown in Fig. 3(b). In Fig. 3(c) we show that both clean and adversarial EEG signals were synchronized with the standard 8 Hz sinusoidal signal, indicated by the green dash–dot lines. Comparing the 13 Hz sinusoidal signal with the magnified difference, the synchronization can also be observed, suggesting that the adversarial perturbation template introduced a frequency component matching the attacker character, which was imperceptible to human eyes but powerful enough to mislead the SSVEP speller.

In Fig. 3(d) we show the spectrum analysis of SSVEPs for 40 stimulation frequencies. We averaged the spectra of the benign EEG signals of the same stimulation frequency from all the subjects and all chosen channels, so that background activities can be suppressed. The first row of Fig. 3(d), for benign trials, clearly shows that the visual stimulus, flickering at a stimulation frequency, can evoke SSVEPs of the same frequency and its harmonics. The second row of Fig. 3(d) shows the same property of adversarial trials, whose attacker character was randomly chosen and fixed for each stimulation frequency. We cannot observe noticeable differences between the two rows in Fig. 3(d), demonstrating the challenge in detecting the adversarial perturbation templates.

## CONCLUSION AND DISCUSSION

In this article we have shown that one can generate adversarial EEG perturbation templates for target attacks for both P300 and SSVEP spellers, i.e. deliberately designed tiny perturbations can manipulate an EEG-based BCI speller to output anything the attacker wants with high success rate, demonstrating the vulnerability of BCI spellers. We should emphasize that the attack framework used here is not specific to the victim models used in this article. They may also be utilized to attack many other classifiers in BCIs with little modification.

### Limitations

The current approaches have two limitations: (a) they require some subject-/model-specific EEG trials to construct the adversarial perturbation template; and (b) they need to know the exact timing of the stimulus to achieve the best attack performance. The adversarial attacks could be more dangerous if these limitations are not resolved.

The first limitation may be alleviated by utilizing the transferability of adversarial examples, which was one of the most dangerous properties of adversarial examples. It was first discovered by Szegedy *et al.* [20] in 2014 and further investigated by many others [24,54–56]. The transferability means that adversarial examples generated from one model can also be used to attack another model, which may have a completely different architecture and/or be trained from a different dataset. Thus, it may be possible to construct the adversarial perturbation template from some existing subjects/models and then apply it to a new subject/model. In the online supplementary material we present experimental results on both cross-subject and cross-model transferability of the generated adversarial perturbations.

The second limitation is that the attacker needs to know the precise time synchronization between adversarial perturbation templates and EEG signals. To study how the synchronization time delay affects the attack performance, we show the relationship between the user/attacker scores and the time delay in adding the perturbation template (see Fig. 3 of the online supplementary material). It can be observed that the SSVEP perturbation template was fairly robust to the time delay whereas the P300 adversarial template was sensitive to the synchronization. For the P300 speller, when the time delay increased, the user score increased rapidly while the attacker score decreased rapidly, suggesting that hiding the time synchronization information may help defend against adversarial attacks in the P300 spellers. However, attacks insensitive to the synchronization may also be possible. For example, the idea of ‘adversarial patch’ [57], which is a tiny picture patch that can mislead the classifier when added anywhere to a large picture to be classified, may be used to increase the robustness to the synchronization time delay. Thus, defending against the attackers may not be an easy task.

### Closed-loop BCI application considerations

In a typical closed-loop BCI speller, the user could receive real-time feedback of his/her chosen character from the screen. If the adversarial perturbation constantly misleads the speller and returns wrong characters that do not match the user’s intentional input, the user would most likely stop using the speller. The consequence may not seem serious for a user that has other means of communication; however, for patients with severe impairments that rely on BCI spellers as their sole mean of communication, e.g. ALS patients, either the attacker changes the meaning of their sentences and they cannot do anything at all, or the patients stop responding, misleading doctors/researchers into thinking they are not able to communicate at all. Both consequences can significantly impact the patients.

Although this article focused on adversarial attacks of P300 and SSVEP spellers, P300 and SSVEP are also widely used in neuroergonomics and assessment of cognitive states, e.g. diagnosis of disorder of consciousness patients [58]. The proposed approach can be used to attack these BCI systems with little modification. The adversarial perturbation could also be a serious concern if the BCI system is used in other scenarios, such as automatic driving, wheelchair control or exoskeleton control, where the feedback could be too late and the cost of one step mistake could be fatal. Moreover, the attacker may only start the attack in some critical conditions. The user is completely unprepared, and the consequences could be more disastrous.

Finally, we need to emphasize again that the goal of this study is not to damage EEG-based BCIs. Instead, we aim to demonstrate that serious adversarial attacks to EEG-based BCIs are possible, and hence expose a critical security concern, which has received little attention before. Our future research will develop strategies to defend against such attacks. Meanwhile, we hope our study can attract more researchers’ attention to the security of EEG-based BCIs.

## METHODS

### Attack the P300 speller

The main idea to construct the adversarial perturbation template was to find a universal perturbation that leads the P300 classifier to classify nontarget epochs into target epochs. We calculated the gradients of the loss with respect to the input nontarget EEG epochs and then summed them as the universal perturbation, assuming the decision boundary is linear. Though the victim model includes nonlinear operations, the attack approach still worked surprisingly well.

Let *X* be an EEG trial, *y* be its label (0 for the nontarget and 1 for the target), *f* be the victim model that gives the label probability for each input *X, J*(*X, y, f*) be the loss function (cross-entropy loss in our case), and *D_NT_* be the dataset containing all nontarget epochs in the training set. Then, the overall direction can be computed as

(6) \begin{eqnarray*} \widetilde{P} = \sum _{(X,y)\in D_{NT}}\frac{\nabla _{X}J(X,1-y,f)}{\Vert \nabla _{X}J(X,1-y,f)\Vert _{F}}. \end{eqnarray*}

After obtaining $\widetilde{P}$, we filtered it by a fourth-order Butterworth bandpass filter of [0.1, 15] Hz, extracted the first 350 ms signal, and then normalized it in each channel so that the L2 norm is 1. Denote the result as $\widehat{P}$. Then, the adversarial perturbation *P* was computed as

(7) \begin{eqnarray*} P = \epsilon \cdot \widehat{P}, \end{eqnarray*}

where ε is a constant controlling the energy of the perturbation (ε = 0.5 in our experiments).

**Figure 4.**
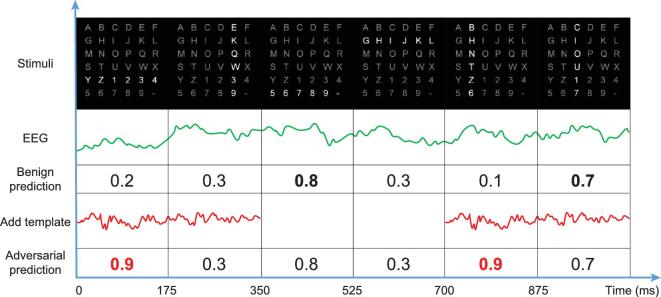
Illustration of the attack procedure in the P300 protocol. The attacker character is *Z*, whereas the user character is *7*. For the benign EEG trial, the P300 speller can correctly identify that P300 is elicited by the intensifications of the last row and the third column. To mislead the P300 speller, an adversarial perturbation template is added during the periods of 0–350 and 700–1050 ms, so that the fifth row and the second column are believed to elicit P300 with the highest probability. The added adversarial perturbation templates do not influence the results of the second and the last stimuli, because their corresponding periods are out of synchronization with the templates. As a result, the P300 speller misclassifies the perturbed trial to attacker character *Z*.

To mislead the P300 speller, one only needs to tamper with some specific signal periods according to the onset of the target stimuli. Because in a practical P300 speller the same row or column is never intensified successively, the perturbation template can last more than one intensification period. In our experiments, the template lasted 2 × 175 = 350 ms, i.e. two intensification periods. In Fig. 4 we illustrate the attack procedure of changing the output from character *7* to character *Z*.

### Attack the SSVEP speller

There are two difficulties in attacking the victim model of the SSVEP speller. First, the victim model is not fixed, as the parameters of the CCA vary in different EEG trials. Second, unlike the P300 speller whose base victim model only needs to classify the input into two classes, there are many more classes in the SSVEP speller. These make adversarial attacks of the SSVEP speller much more challenging.

The remedy was to generate the adversarial perturbation template $\delta _{\widehat{f}}\in \mathbb {R}^{N_e \times N_s}$, which can lead the SSVEP speller to output the attacker character of stimulation frequency $\widehat{f}$. For each user, we used the first block $\mathcal {D}=\lbrace X_i\rbrace _{i=1}^{N}$ to craft $\delta _{\widehat{f}}$, and the remaining five blocks to evaluate its attack performance.

According to the victim model, $\delta _{\widehat{f}}$ should be able to maximize $\rho (X+\delta _{\widehat{f}},Y_{\widehat{f}})$ in equation (4), such that

(8) \begin{eqnarray*} \arg \max _{f\in F} \rho (X+\delta _{\widehat{f}},Y_f) = \widehat{f}. \end{eqnarray*}

To simplify the optimization and ensure the integrity of the adversarial template during signal filtering, we show in the online supplementary material that the problem can be converted to

(9) \begin{eqnarray*} \min _{\mathbf {r}_{\widehat{f}}} &-& \sum _{X\in \mathcal {D}} \mbox{tr}(S(X+\mbox{filt}(\mathbf {r}_{\widehat{f}}), Y_{\widehat{f}}))\nonumber\\ &&+\,\,\alpha \cdot \Vert \mbox{filt}(\mathbf {r}_{\widehat{f}})\Vert _F, \end{eqnarray*}

where *S*(*X, Y*) is defined in equation (3), filt(·) means retaining only the 7–90 Hz effective signal frequency components and $\alpha \cdot \Vert \mbox{filt}(\mathbf {r}_{\widehat{f}})\Vert _F$ penalizes the energy of the perturbation.

Gradient descent was used to update $\mathbf {r}_{\widehat{f}}$, and then $\delta _{\widehat{f}}=\mbox{filt}(\mathbf {r}_{\widehat{f}})$. The iteration stopped when the SPR was lower than a threshold, which was set to 25 dB in our experiments.

## DATA AVAILABILITY STATEMENT

Publicly available BCI datasets were used in this study. The P300 speller dataset can be downloaded from http://www.bbci.de/competition/iii/#data_set_ii (Dataset II) [45]. The P300 speller dataset of ALS patients was first used in [59] and can be downloaded from http://bnci-horizon-2020.eu/database/data-sets (P300 speller with ALS patients (008-2014)). The SSVEP dataset can be downloaded from http://bci.med.tsinghua.edu.cn/download.html [19]. All source code is available on GitHub (https://github.com/ZhangXiao96/Speller-Attacks).

## Supplementary Material

nwaa233_Supplemental_File
