# Supplementary material for: Tiny noise, big mistakes: adversarial perturbations induce errors in brain–computer interface spellers
Source: Natl Sci Rev. 2020 Sep 10;8(4):nwaa233. doi: 10.1093/nsr/nwaa233 (PMC8288388; doi:10.1093/nsr/nwaa233)
Supplement: nwaa233_Supplemental_File [file nwaa233_supplemental_file.pdf]

National Science Review
?: 1–?, 2020
doi: ??????????
Advance access publication ? ? 2020

RESEARCH ARTICLE

INFORMATION SCIENCE

Supplementary Information
Tiny noise, big mistakes: adversarial perturbations induce errors in Brain-Computer Interface spellers

Xiao Zhang[1], Dongrui Wu[1,*], Lieyun Ding[2,*], Hanbin Luo[2], Chin-Teng Lin[3], Tzyy-Ping Jung[4,5] and Ricardo Chavarriaga[6]

[1]Ministry of Education Key Laboratory of Image Processing and Intelligent Control, School of Artificial Intelligence and Automation, Huazhong University of Science and Technology, Wuhan 430074, China;
[2]School of Civil Engineering and Mechanics, Huazhong University of Science and Technology, Wuhan 430074, China;
[3]Centre of Artificial Intelligence, Faculty of Engineering and Information Technology, University of Technology Sydney, Sydney 2007, Australia;
[4]Swartz Center for Computational Neuroscience, Institute for Neural Computation, University of California San Diego (UCSD), La Jolla, CA 92093, USA;
[5]Center for Advanced Neurological Engineering, Institute of Engineering in Medicine, UCSD, La Jolla, CA 92093, USA;
[6]ZHAW DataLab, Zürich University of Applied Sciences, Winterthur 8401, Switzerland.
*Corresponding authors. E-mails: drwu@hust.edu.cn; dly@hust.edu.cn

ABSTRACT
This is the supplementary information of the manuscript "Tiny noise, big mistakes: adversarial perturbations induce errors in Brain-Computer Interface spellers".

Keywords: electroencephalogram, brain-computer interfaces, BCI spellers, adversarial examples

THE VICTIM MODEL OF THE P300 SPELLER

The details of the victim model of the P300 speller are introduced.

xDAWN spatial filters

The original xDAWN filter [1] was designed for P300 evoked potentials by enhancing the target response with respect to the non-target response. We used a generalized version in our experiments, which was implemented in *pyRiemann*[1].

More specifically, let $\mathbf{D} = \{(X_i, y_i)\}_{i=1}^N$ be the training set, where $X_i \in \mathbb{R}^{N_e \times N_s}$ is the i-th mean-centered EEG epoch (N_e is the number of channels, and N_s the number of time domain samples), and $y_i \in \{0, 1\}$ its corresponding label (0 for *non-target*, and 1 for *target*). The average epoch $\overline{X}_c$, $c \in \{0, 1\}$, is first calculated. Spatial filters $U_c \in \mathbb{R}^{N_f \times N_e}$ were then designed to maximize the signal to signal-plus-noise ratio for each class:

$$U_c = \arg\max_U \frac{\mathrm{tr}\left(U\overline{X}_c\overline{X}_c^T U^T\right)}{\mathrm{tr}\left(U X_{all} X_{all}^T U^T\right)}, \tag{1}$$

where N_f is the number of filters ($N_f = 8$ was used in our experiments), X_{all} is obtained by concatenating all EEG epochs in $\mathbf{D}$ along the channels, and tr is the trace of a matrix. Generalized eigenvalue decomposition can be used to solve equation (1).

[1]https://pyriemann.readthedocs.io/en/latest/index.html

After obtaining the filters for both classes, the concatenated spatial filters $U = [U_0; U_1]$ can be used to filter each EEG epoch:

$$\widetilde{X}_i = UX_i. \tag{2}$$

Tangent space projection

Covariance matrices of the EEG trials are widely-used in BCIs. However, they lie on a Riemannian manifold of Symmetric Positive Definite (SPD) matrices, and hence cannot be directly used by Euclidean space classifiers, such as Logistic Regression and Support Vector Machines. To solve this problem, the covariance matrices are projected onto the Euclidean tangent space of a reference SPD matrix, and then the vectorized features are used by Euclidean space classifiers.

More specifically, we first calculate the augmented covariance matrix C_i for each $\widetilde{X}_i$:

$$C_i = \begin{bmatrix} ZZ^T, & Z\widetilde{X}_i^T \\ \widetilde{X}_i Z^T, & \widetilde{X}_i \widetilde{X}_i^T \end{bmatrix}, \tag{3}$$

where $Z = \left[U\overline{X}_0; U\overline{X}_1\right]$. Then, C_i is projected onto the tangent space of the reference SPD matrix C_f, which is the geometric mean of $\{C_i\}_{i=1}^N$, i.e.,

$$C_f = \arg\min_C \left(\sum_{i=1}^N \delta(C, C_i)^2 \right), \tag{4}$$

where $\delta(C_A, C_B)$ is the Affine Invariant Riemannian Metric distance:

$$\delta(C_A, C_B) = \left\| \text{logm}\left(C_A^{-1/2} C_B C_A^{-1/2} \right) \right\|. \tag{5}$$

The vectorized features are:

$$\mathbf{s}_i = \text{upper}\left(\text{logm}\left(C_f^{-1/2} C_i C_f^{-1/2} \right) \right), \tag{6}$$

where upper$(\cdot)$ vectorizes the upper triangular part of a symmetric matrix. A weight of $\sqrt{2}$ is applied to the off-diagonal elements, and a weight of 1 to the rest, during the vectorization. $\mathbf{s}_i$ can then be fed into any Euclidean space classifier.

CANONICAL CORRELATION ANALYSIS (CCA)

This section introduces CCA, which can be used to extract the underlying correlation between two time series.

Problem setup

Let $X \in \mathbb{R}^{C_1 \times N}$ and $Y \in \mathbb{R}^{C_2 \times N}$ be two multi-channel time series, where C_1 and C_2 represent the number of channels, and N the number of time domain samples. X and Y are z-normalized in each channel.

The main idea of CCA is to find a pair of canonical variables, denoted as $\mathbf{a} \in \mathbb{R}^{C_1 \times 1}$ and $\mathbf{b} \in \mathbb{R}^{C_2 \times 1}$, for X and Y respectively, so that the correlation coefficient ρ between $\mathbf{a}^T X$ and $\mathbf{b}^T Y$ can be maximized. The problem can be mathematically formulated as:

$$\max_{\mathbf{a},\mathbf{b}} \frac{\mathbf{a}^T XY^T \mathbf{b}}{\sqrt{\mathbf{a}^T XX^T \mathbf{a}} \sqrt{\mathbf{b}^T YY^T \mathbf{b}}}, \tag{7}$$

which can be re-expressed as:

$$\begin{aligned} \max_{\mathbf{a},\mathbf{b}} \quad & \mathbf{a}^T XY^T\mathbf{b}, \\ \text{s.t.} \quad & \mathbf{a}^T XX^T\mathbf{a} = 1, \mathbf{b}^T YY^T\mathbf{b} = 1. \end{aligned} \tag{8}$$

Solution of CCA

There are several approaches to solve equation (8). Here we introduce the Lagrange multiplier method [2].

Denote AB^T by S_{AB}. Then, equation (8) can be rewritten as:

$$\begin{aligned} \max_{\mathbf{a},\mathbf{b}} \quad & \mathbf{a}^T S_{XY}\mathbf{b}, \\ \text{s.t.} \quad & \mathbf{a}^T S_{XX}^T\mathbf{a} = 1, \mathbf{b}^T S_{YY}^T\mathbf{b} = 1. \end{aligned} \tag{9}$$

According to the Lagrange multiplier method, equation (9) is equivalent to $\max_{\mathbf{a},\mathbf{b},\lambda,\theta} J(\mathbf{a},\mathbf{b},\lambda,\theta)$, where:

$$J(\mathbf{a},\mathbf{b},\lambda,\theta) = \mathbf{a}^T S_{XY}\mathbf{b} - \frac{\lambda}{2}(\mathbf{a}^T S_{XX}\mathbf{a} - 1) - \frac{\theta}{2}(\mathbf{b}^T S_{YY}\mathbf{b} - 1). \tag{10}$$

By setting the first partial derivatives to zero, i.e.,

$$\nabla_{\mathbf{a}} J = S_{XY}\mathbf{b} - \lambda \cdot S_{XX}\mathbf{a} = 0, \tag{11}$$

$$\nabla_{\mathbf{b}} J = S_{YX}\mathbf{a} - \theta \cdot S_{YY}\mathbf{b} = 0, \tag{12}$$

$$\frac{\partial J}{\partial \lambda} = -\frac{1}{2}(\mathbf{a}^T S_{XX}\mathbf{a} - 1) = 0, \tag{13}$$

$$\frac{\partial J}{\partial \theta} = -\frac{1}{2}(\mathbf{a}^T S_{YY}\mathbf{b} - 1) = 0, \tag{14}$$

we have

$$\lambda = \theta = \mathbf{a}^T S_{XY}\mathbf{b}. \tag{15}$$

It should be noted that equation (15) is also the definition of the correlation coefficient ρ.

According to equations (11) and (12), we have:

$$S_{XX}^{-1}S_{XY}S_{YY}^{-1}S_{YX}\mathbf{a} = \lambda^2\mathbf{a} = \rho^2\mathbf{a}, \tag{16}$$

which implies that ρ^2 equals the largest eigenvalue of $S_{XX}^{-1}S_{XY}S_{YY}^{-1}S_{YX}$, and $\mathbf{a}$ is the corresponding eigenvector. $\mathbf{b}$ can be obtained in a similar way.

DETAILS OF THE ATTACK METHOD FOR THE SSVEP SPELLER

As shown in the main body of our paper, the key to craft $\delta_{\widehat{f}}$ is to solve

$$\arg\max_{f\in F} \rho(X + \delta_{\widehat{f}}, Y_f) = \widehat{f}. \tag{17}$$

In other words, $\delta_{\widehat{f}}$ can be crafted by solving

$$\max_{\delta_{\widehat{f}}} \sum_{X\in\mathcal{D}} \lambda_{\max}(S(X + \delta_{\widehat{f}}, Y_{\widehat{f}})). \tag{18}$$

Since $S(X+\delta_{\hat{f}}, Y_f)$ is not symmetric, it is difficult to calculate the derivatives of its largest eigenvalue, resulting in challenges in optimization. Considering the fact that the largest eigenvalue is always no smaller than the average of all eigenvalues:

$$\begin{aligned}\lambda_{\max}(S(X+\delta_{\hat{f}}, Y_f)) &\geqslant \frac{1}{N_e}\sum\nolimits_j \lambda_j(S(X+\delta_{\hat{f}}, Y_f)) \\ &= \frac{1}{N_e}\mathrm{tr}(S(X+\delta_{\hat{f}}, Y_f)),\end{aligned} \tag{19}$$

instead of solving equation (18) directly, we can maximize its lower bound to reduce the optimization difficulty:

$$\max_{\delta_{\hat{f}}} \sum_{X\in\mathcal{D}} \mathrm{tr}(S(X+\delta_{\hat{f}}, Y_{\hat{f}})). \tag{20}$$

Because the effective frequency band of SSVEP signals is 7-90 Hz, we introduced a new variable $\mathbf{r}_{\hat{f}}$ so that

$$\delta_{\hat{f}} = \mathrm{filt}(\mathbf{r}_{\hat{f}}), \tag{21}$$

where filt$(\cdot)$ means retaining only the 7-90 Hz effective signal frequency components. As a result, we can ensure the integrity of the adversarial template during signal filtering. In addition, we added $\alpha \cdot \|\delta_{\hat{f}}\|_F$ to penalize the energy of the perturbation, where α is the penalty coefficient.

Finally, the problem becomes:

$$\min_{\mathbf{r}_{\hat{f}}} \; -\sum_{X\in\mathcal{D}} \mathrm{tr}(S(X+\mathrm{filt}(\mathbf{r}_{\hat{f}}), Y_{\hat{f}})) + \alpha \cdot \|\mathrm{filt}(\mathbf{r}_{\hat{f}})\|_F. \tag{22}$$

SECURITY OF P300 SPELLER FOR AMYOTROPHIC LATERAL SCLEROSIS (ALS) PATIENTS

We performed additional experiments to investigate how adversarial perturbations impact ALS patients on P300 Spellers [3]. The eight-channel (Fz, Cz, Pz, Oz, P3, P4, PO7 and PO8) EEG signals were recorded from eight ALS patients. The EEG data were digitized at 256 Hz, bandpass filtered to 0.1-30 Hz, and then z-normalized for each channel. For each subject, there were 21 characters for training and 14 for testing. Each character corresponds to a set of 12 random intensifications, which were repeated 20 times. Each intensification lasted for 125 ms, followed by a 125 ms blank. In our experiments, 10 repeats were utilized to output a character during the test.

We applied the same Riemannian geometry based approach to recognizing the existence of P300 potentials. The only difference from the previous study was that the number of xDAWN spatial filters was eight. As shown in the 'Before attack' panel of Table 1, the victim models demonstrated good performance without attacks, and also high robustness to Gaussian noise perturbations. However, the 'After attack' panel shows that all user scores and ITRs were more or less reduced after adversarial perturbations. For half of the subjects (subjects 1, 2, 5 and 7), the user scores and ITRs approached zero, i.e., the P300 speller became almost completely useless, indicating a serious security concern of P300 spellers to ALS patients.

TRANSFERABILITY OF ADVERSARIAL PERTURBATIONS

We have mentioned that one limitation of the attack approaches is that they require some subject-/model- specific information to construct adversarial perturbation templates. One possible solution to alleviate this problem is to enhance the transferability of adversarial perturbations: the attacker can generate adversarial perturbations based on EEG signals gathered by himself/herself, or any model he/she chooses to use, and then utilize them to attack another

Table 1 P300 speller attack results for eight ALS patients. Before attack: Baselines on clean EEG data (without adding any perturbations) and Gaussian-noise-perturbed EEG data, and the corresponding SPRs (dB). After attack: Average user/attacker scores/ITRs of the 36 attacker characters in target attacks, and the corresponding period and trial SPRs (dB). $\epsilon = 0.8$ for all the perturbations.

Sub.	Before attack					After attack					
	Clean		Gaussian noise			User		Attacker		Period	Trial
	Score	ITR	Score	ITR	SPR	Score	ITR	Score	ITR	SPR	SPR
1	0.79	6.57	0.79	6.57	22.6	0.03	0.04	1.00	10.22	22.6	27.4
2	0.93	8.76	0.93	8.76	22.4	0.10	0.26	0.74	6.09	22.4	27.5
3	1.00	10.22	1.00	10.22	22.9	0.53	3.59	0.17	0.67	22.9	27.7
4	1.00	10.22	0.93	8.76	23.1	0.45	2.83	0.22	1.10	23.1	27.9
5	1.00	10.22	1.00	10.22	22.2	0.05	0.12	0.86	7.72	22.2	27.1
6	0.93	8.76	0.86	7.60	22.4	0.21	0.86	0.44	2.75	22.4	27.2
7	1.00	10.22	1.00	10.22	22.9	0.03	0.05	0.96	9.45	22.9	27.7
8	1.00	10.22	1.00	10.22	23.1	0.98	9.73	0.03	0.05	23.1	28.0

subject/model. Here we present some experimental results on the cross-subject and cross-model transferability of our adversarial perturbations (for P300 spellers, the ALS patient dataset was used due to its larger number of subjects). We found that adversarial perturbations for SSVEP spellers seem to have better transferability than P300 spellers.

Cross-subject transferability

We used adversarial perturbations generated from one subject to attack the victim model of another subject. Figure 1 shows the average attacker scores of cross-subject attacks. There was almost no cross-subject transferability of adversarial perturbation templates for P300 spellers, whereas perturbations for SSVEP spellers can usually successfully attack victim models of different subjects. Additionally, some subjects were much more robust to transfer attacks, e.g., Subjects 12, 22 and 25 in Figure 1b.

Why adversarial perturbation templates demonstrated poor cross-subject transferability for P300 spellers will be investigated in more depth in our future research.

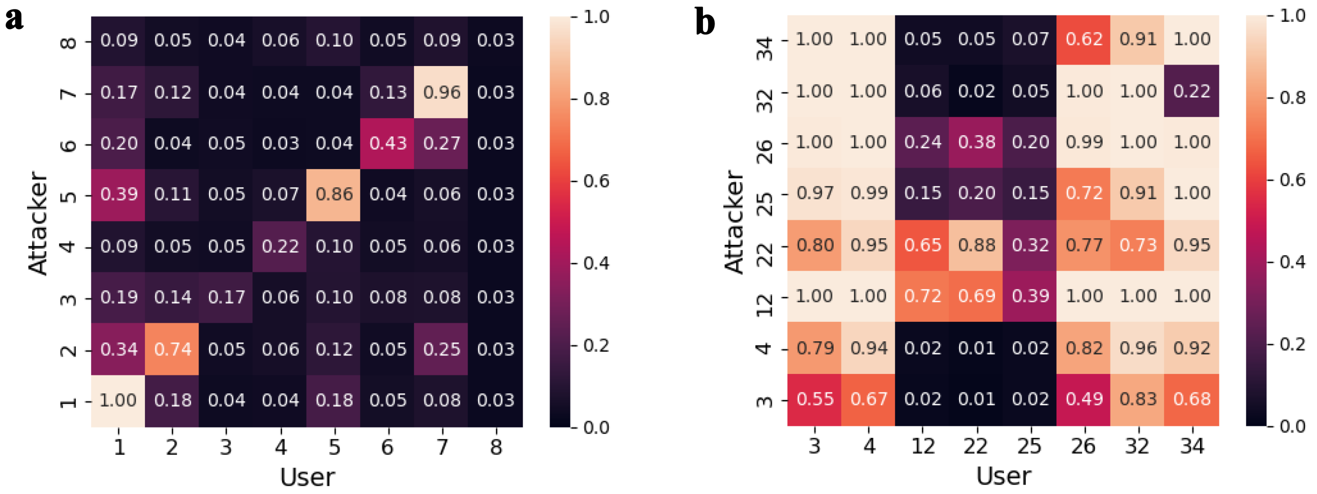

Figure 1 Cross-subject transferability of adversarial perturbations. The heatmap shows the average attacker scores when using the adversarial perturbations of one subject to attack another subject. **a**, attacker scores for the P300 speller. **b**, attacker scores for the SSVEP speller.

Cross-model transferability

Cross-model transferability requires adversarial perturbations to be able to attack different EEG classification pipelines, which means the attacker does not need access to victim models any more, implying a more serious threat to the security of BCI spellers. This subsection presents the attack performance of our generated adversarial perturbations on new EEG classification pipelines.

For P300 spellers, the new classification pipeline consisted of xDAWN filtering and Logistic Regression classification, and the adversarial perturbation templates were again generated from the Riemannian geometry based approach. The 'Before attack' panel of Table 2 shows that the new pipeline had high classification accuracy without attacks, and it was also robust to Gaussian noise. The 'After attack' panel shows that the new pipeline can still be manipulated by adversarial perturbation templates constructed from a different pipeline, though not as much as that in Table 1. Comparing the attack performances in Tables 1 and 2, it seems that an adversarial perturbation template with better attack performance on the model it was generated from may also have better cross-model transferability to attack another model.

Table 2 P300 speller cross-model attack results for eight ALS patients. The victim model (xDAWN and Logistic Regression) was different from the attacker model (a Riemannian geometry based approach), based on which adversarial perturbations were generated. Before attack: Baselines on clean EEG data (without adding any perturbations) and Gaussian-noise-perturbed EEG data, and the corresponding SPRs (dB). After attack: Average user/attacker scores/ITRs of the 36 attacker characters in target attacks, and the corresponding period and trial SPRs (dB). $\epsilon = 0.8$ for all perturbations.

Sub.	Before attack					After attack					
	Clean		Gaussian noise			User		Attacker		Period	Trial
	Score	ITR	Score	ITR	SPR	Score	ITR	Score	ITR	SPR	SPR
1	0.86	7.60	0.86	7.60	22.6	0.03	0.04	1.00	10.22	22.6	27.4
2	1.00	10.22	1.00	10.22	22.4	0.53	3.56	0.24	0.99	22.4	27.5
3	1.00	10.22	1.00	10.22	22.9	0.62	4.66	0.15	0.60	22.9	27.7
4	0.79	6.57	0.79	6.57	23.1	0.49	3.21	0.20	0.91	23.1	27.9
5	0.86	7.60	0.86	7.60	22.2	0.17	0.60	0.60	4.31	22.2	27.1
6	1.00	10.22	1.00	10.22	22.4	0.36	1.90	0.31	1.66	22.4	27.2
7	1.00	10.22	1.00	10.22	22.9	0.17	0.61	0.53	3.59	22.9	27.7
8	1.00	10.22	1.00	10.22	23.1	0.94	9.04	0.04	0.06	23.1	28.0

For SSVEP spellers, we utilized Filter Bank Canonical Correlation Analysis (FBCCA)[2] as our new victim model [4]. Table 3 shows the baseline performance of FBCCA and the attack performance of adversarial perturbations (generated from CCA) on this model. FBCCA demonstrated promising performance on clean and randomly perturbed EEG signals. However, adversarial perturbations generated from CCA can still manipulate the output characters of FBCCA, verifying that cross-model transferability also exists in SSVEP spellers.

The key of the transferability is to find the most common patterns shared by different models, hence the adversarial perturbations affecting these patterns can attack as many models as possible. From this point of view, generating adversarial perturbations based on the ensemble of multiple models seems to be a promising direction. We will explore this in our future research.

ADDITIONAL FIGURES

Figure 2 presents the baseline performances of all 35 subjects for the SSVEP dataset. Figure 3 shows how the synchronization time delay affects the attack performance.

[2]Our implementation was adapted from `https://github.com/hisunjiang/CCAforSSVEP`.

Table 3 SSVEP speller cross-model attack results. The victim model (FBCCA) was different from the attacker model (CCA), based on which adversarial perturbations were generated. Before attack: Baselines on clean data (without adding any perturbations), Gaussian-noise-perturbed EEG data and periodic-noise-perturbed EEG data (single/compound). After attack: Average user/attacker scores/ITRs of 40 attacker characters in target attacks, and the corresponding SPRs (dB).

Sub.	Before attack							After attack				
	Clean		Gaussian Noise		S/C Periodic Noise		SPR	User		Attacker		SPR
	Score	ITR	Score	ITR	Score	ITR		Score	ITR	Score	ITR	
3	0.98	218.7	0.98	219.0	0.96/0.97	212.7/217.7	25.0	0.06	2.3	0.92	204.2	25.3
4	0.88	181.4	0.88	182.2	0.84/0.88	169.0/180.1	25.0	0.03	0.0	1.00	230.6	25.7
12	0.85	173.3	0.82	163.1	0.81/0.81	159.9/159.9	25.0	0.05	2.2	0.97	219.0	25.5
22	0.95	207.4	0.95	208.1	0.94/0.95	204.7/206.4	25.0	0.03	0.4	0.99	228.6	25.1
25	0.87	176.8	0.84	168.1	0.83/0.84	163.4/166.4	25.0	0.10	7.8	0.90	194.0	26.7
26	0.87	177.9	0.86	174.1	0.75/0.82	139.8/159.6	25.0	0.03	0.0	1.00	231.4	24.8
32	0.88	181.2	0.87	180.1	0.80/0.85	157.1/170.5	25.0	0.03	0.0	1.00	231.4	24.9
34	0.88	180.7	0.85	171.3	0.72/0.80	132.6/155.9	25.0	0.03	0.0	0.98	226.4	25.9

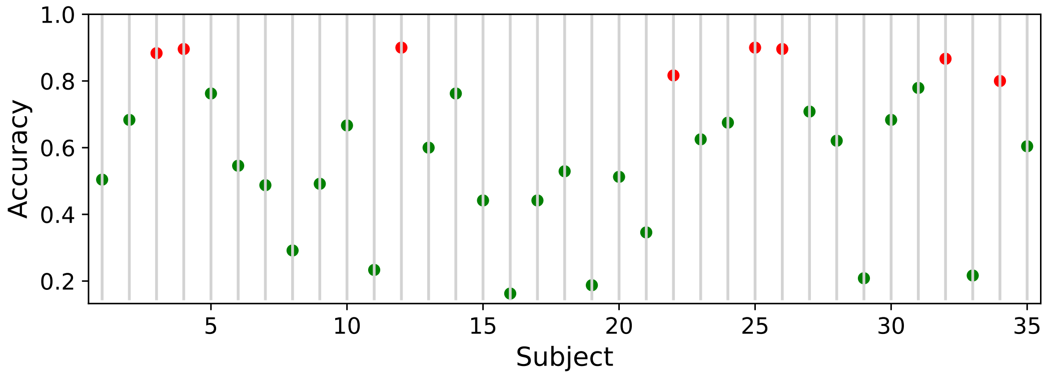

Figure 2 Classification accuracies of all 35 subjects for the clean SSVEP dataset. CCA was utilized to recognize the user characters. Eight subjects (3, 4, 12, 22, 25, 26, 32, 34) with the best baseline performances are shown in red, whereas the others in green.

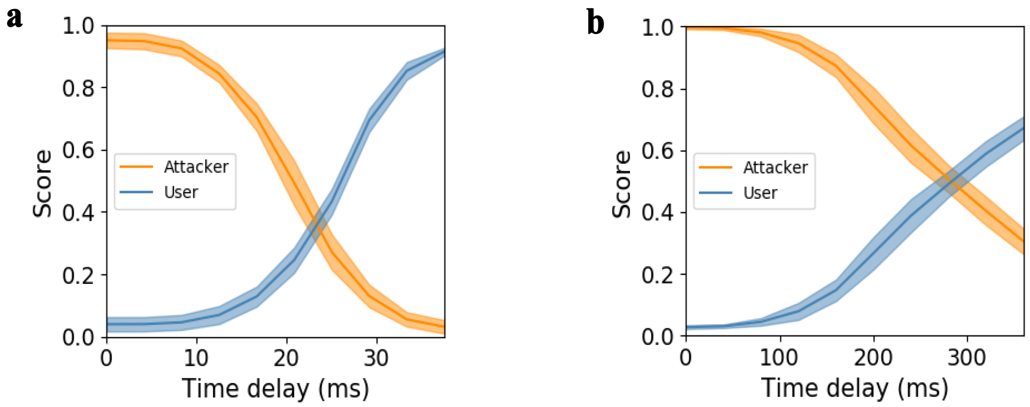

Figure 3 User and attacker scores with respect to the synchronization time delay. The curve represents the mean of all attacker characters, and the shadow the standard deviation. **a**, scores for the P300 speller, where 100 test trials for Subject A were perturbed to be misclassified as each of the 36 attacker characters. **b**, scores for the SSVEP speller, where $5 \times 40 = 200$ test trials for Subject 26 were perturbed to be misclassified as each of the 40 attacker characters.

REFERENCES

1. Rivet B, Souloumiac A and Attina V *et al.* xDAWN algorithm to enhance evoked potentials: application to brain-computer interface. *IEEE Trans Biomed Eng* 2009; **56**: 2035–2043.
2. Hestenes MR. Multiplier and gradient methods. *J Optim Theory Appl* 1969; **4**: 303–320.
3. Riccio A, Simione L and Schettini F *et al.* Attention and P300-based BCI performance in people with amyotrophic lateral sclerosis. *Front Hum Neurosci* 2013; **7**: 732.
4. Chen X, Wang Y and Gao S *et al.* Filter bank canonical correlation analysis for implementing a high-speed SSVEP-based brain–computer interface. *J Neural Eng* 2015; **12**: 046008.
